# Sub-Saharan Africa preparedness and response to the COVID-19 pandemic: A perspective of early career African scientists

**DOI:** 10.12688/wellcomeopenres.16070.3

**Published:** 2020-12-10

**Authors:** Gisele Umviligihozo, Lucy Mupfumi, Nelson Sonela, Delon Naicker, Ekwaro A. Obuku, Catherine Koofhethile, Tuelo Mogashoa, Anne Kapaata, Geoffrey Ombati, Clive M. Michelo, Kimani Makobu, Olamide Todowede, Sheila N. Balinda

**Affiliations:** 1 Faculty of Health Sciences, Simon Fraser University, Burnaby, British Columbia, V5A 1S6, Canada; 2 Botswana Harvard AIDS Institute Partnership (BHP), Private Bag BO 320, Bontleng, Gaborone, Botswana; 3 Chantal Biya International Reference Center for research on the management and prevention of HIV/AIDS (CIRCB), Yaoundé, B.P.: 3077, Cameroon; 4 School of Medicine, Physical and Natural Sciences, University of Rome Tor Vegata, Rome, 1-00133, Italy; 5 HIV Pathogenesis Programme, The Doris Duke Medical Research Institute, Nelson R. Mandela, School of Medicine, University of KwaZulu-Natal, Durban, 4001, South Africa; 6 CoVID Pandemic Rapid Evidence Synthesis Group (CoVPRES), Africa Centre for Systematic Reviews and Knowledge Translation, College of Health Sciences, Makerere University, Kampala, P.O. Box 7072, Uganda; 7 Harvard T.H. Chan School of Public Health, Boston, 651 Huntington Ave, Boston, MA, 02115, USA; 8 International AIDS Vaccine Initiative (IAVI)- Vaccine, Immunology, Science and Technology for Africa (VISTA), Medical Research Council (MRC)/ (Uganda Virus Research Institute) UVRI & London School of Hygiene and Tropical Medicine (LSHTM) Uganda Research Unit, Entebbe, P.O.Box 49, Uganda; 9 Kenya AIDS Vaccine Initiative-Institute of Clinical Research (KAVI-ICR), College of Health Sciences, University of Nairobi, Nairobi, P.O. Box 19676, Kenya; 10 Rwanda Zambia HIV Research Group (RZHRG), Kigali Rwanda, Lusaka and Ndola, PostNet 412, P/Bag E891, Zambia; 11 Kenya Medical Research Institute (KEMRI), Wellcome Trust Research Program (KWTRP), Kilifi, P.O Box 230, Kenya; 12 Center for Health Services Studies, University of Kent, Canterbury, CT2 7NF, UK; 13 School of Nursing and Public Health, College of Health Sciences, University of KwaZulu Natal, Durban, 4001, South Africa

**Keywords:** Sub-Saharan Africa, SARS-CoV-2, COVID-19, pandemic, preparedness, and response.

## Abstract

Emerging highly transmissible viral infections such as SARS-CoV-2 pose a significant global threat to human health and the economy. Since its first appearance in December 2019 in the city of Wuhan, Hubei province, China, SARS-CoV-2 infection has quickly spread across the globe, with the first case reported on the African continent, in Egypt on February 14 ^th^, 2020. Although the global number of COVID-19 infections has increased exponentially since the beginning of the pandemic, the number of new infections and deaths recorded in African countries have been relatively modest, suggesting slower transmission dynamics of the virus on the continent, a lower case fatality rate, or simply a lack of testing or reliable data. Notably, there is no significant increase in unexplained pneumonias or deaths on the continent which could possibly indicate the effectiveness of interventions introduced by several African governments. However, there has not yet been a comprehensive assessment of sub-Saharan Africa’s (SSA) preparedness and response to the COVID-19 pandemic that may have contributed to prevent an uncontrolled outbreak so far. As a group of early career scientists and the next generation of African scientific leaders with experience of working in medical and diverse health research fields in both SSA and resource-rich countries, we present a unique perspective on the current public health interventions to fight COVID-19 in Africa. Our perspective is based on extensive review of the available scientific publications, official technical reports and announcements released by governmental and non-governmental health organizations as well as from our personal experiences as workers on the COVID-19 battlefield in SSA. We documented public health interventions implemented in seven SSA countries including Uganda, Kenya, Rwanda, Cameroon, Zambia, South Africa and Botswana, the existing gaps and the important components of disease control that may strengthen SSA response to future outbreaks.

## Disclaimer

The views expressed in this article are those of the author(s) and do not reflect those of their employers or institutions. Publication in Wellcome Open Research does not imply endorsement by Wellcome.

## Introduction

COVID-19 is caused by a novel beta-coronavirus named severe acute respiratory syndrome coronavirus 2 (SARS-CoV-2) that was first reported in December 2019 in the city of Wuhan, Hubei province, China ^1^. SARS-CoV-2 infection has quickly spread across the globe ^2^, with the first case reported on the African continent, in Egypt on February 14 ^th^ 2020 ^3^. COVID-19 was declared a public health emergency of international concern on January 30 ^th^ 2020 and a pandemic on March 11 ^th^ 2020 by the World Health Organization (WHO) ^4, 5^. Just a few months into the pandemic, COVID-19 has ravaged developed countries, with significant case fatality rates in Europe and the USA ^6^. Considering the large number of people that live in poor and crowded informal settings in sub-Saharan Africa (SSA) ^7, 8^, the mode of transmission of SARS-CoV-2, the severity of the disease as well as the existing fragile health systems ^9– 11^ it was hypothesized that Africa may markedly be affected by the COVID-19 pandemic resulting in disastrous consequences with a large number of patients overwhelming hospitals and high case fatality rates ^9, 12^.

As of June 6 ^th^ COVID-19 global infections recorded in 215 countries were 6,663,034 cases and 392,802 deaths [5.9%]. The three countries with the highest burden were the USA with 1,857,872 cases [deaths 5.8%], Brazil 614,941 [5.5%] and Russia 458,689 [1.2%]. Italy and Spain were highly affected in the first quarter of the year but have stabilised their epidemics since the beginning of May 2020 with 234,531 cases [deaths: 14.4%] and 240,978 [11.3%] respectively ^6^. At the same time, the African continent accounted for only 2.6% of the global infections with 177,953 cases and 4,936 deaths [2.8%] in 54 countries ^13^. Four African countries, South Africa, Egypt, Morocco and Algeria, comprise more than 52% of reported COVID-19 cases in Africa ^13^ and only one of these countries is located in the SSA region. Although the global number of SARS-CoV-2 infections increased exponentially since the beginning of the pandemic due to ongoing transmission, the low number of infections and deaths recorded in SSA countries have raised suspicions on whether they represent a slow progression of the pandemic in Africa, a lower case fatality rate, or simply a lack of testing or reliable data. Notably, there is no significant increase in unexplained pneumonias or deaths recorded on the continent which could possibly indicate the effectiveness of interventions introduced by several African governments, although recent reports from South Africa indicate a surge in weekly deaths from natural causes that may be attributable to COVID-19 ^14^. However, there has not yet been a comprehensive assessment of SSA’s preparedness and response to the COVID-19 pandemic that may have contributed to prevent an uncontrolled outbreak so far.

Here, we – a diverse group of early career researchers (graduate students and post-doctoral scientists) and the next generation of African scientific leaders with experience working in various fields of health research including medicine, immunology, molecular biology, microbiology, virology and public health in both SSA countries and in developed countries – conducted an assessment of African preparedness and response to the COVID-19 during the early (first 3 months) of the pandemic on the African continent. The early career researchers involved are fellows of the African Academy of Science’s Sub-Saharan African Network for TB/HIV Research Excellence (SANTHE) ^15^, (part of the DELTAS Africa initiative) and some were at the frontline of the battle against COVID-19 in their respective countries during the time of this assessment.

As of June 6 ^th^ 2020, the seven countries represented had reported the following caseloads of COVID-19: Cameroon, 7,599 cases [deaths: 2.8%]; Kenya, 2,600 [3.2%]; Zambia, 1,089 [0.6%]; Rwanda, 431 [0.5%]; Uganda, 593 [0%]; Botswana, 40 [2.5%]; ^13^ and South Africa, which had recorded the highest number of COVID-19 cases in Africa with 45,973 cases [2.1% deaths] ^13^. Even though the COVID-19 pandemic unfolded rapidly, and the undertaken public health measures interrupted our studies, our careers and other usual activities, this pandemic has also offered a blueprint on how to deal with epidemics. This analysis presents a unique perspective on the currently developed public health interventions to fight COVID-19 on the African continent. We discuss the challenges and opportunities that exist to improve African capacity to fight future epidemics from our perspective as the next generation of scientists that will oversee these responses in the future.

Our evaluation of sub-Saharan Africa preparedness and response to COVID-19 reviewed country specific preventive measures and critically examined the response to the pandemic in seven African countries. We assessed the public health measures and other crucial interventions that were put in place in the control of COVID-19 in Uganda, Kenya, Rwanda, Cameroon, Zambia, South Africa and Botswana. We argue that these strategies may have helped to prevent a disastrous outcome by reducing rapid transmissions that may happen in clusters and minimizing the number of patients seeking medical assistance at the same time. We also identified and summarized in three categories of biomedical, sociocultural and economic factors; the challenges encountered at different levels of the health system. We presented the gaps existing in the public health intervention programs that may result in delays/failure to halt the spread of the disease as well as the important components of disease control that may strengthen sub-Saharan Africa preparedness and response to future outbreaks.

### Assessment and findings

The rapid rise of SARS-CoV-2 infections sent a clear message to the world that quick action was needed to prevent the spread of the disease. WHO warned on February 22 ^nd^ 2020 that all member states of the African Union should develop an early strong plan of action to tackle the growing threat ^16^. As emerging African leaders in health research and fellows of the SANTHE consortium, we convened a virtual meeting to discuss the threat that the pandemic posed to SSA and agreed that we had a responsibility to critically analyze the response of our governments so far and to offer our own perspective on how this and other similar epidemics should be tackled on the continent. In the meeting, participants discussed the origin of the new SARS-CoV-2 virus, infection preventive measures, diagnostic tests, clinical management of the disease and the development of vaccines and therapeutics by critically reviewing the available scientific literature. We discussed the need for scientific and evidence-based responses that considered Africa’s unique healthcare, sociocultural and economic challenges. We therefore decided to review articles focusing on SARS-CoV-2 infection dynamics in Africa and the responses that African governments were taking. Specifically, we used the free search engine PubMed to identify articles that discussed the transmission dynamics of the new SARS-CoV-2 virus, or described the sociocultural, economic and the state of health care systems in Africa and their impact on SARS-CoV-2 transmission. Moreover, we searched for popular media reports and performed internet searches at official websites for any documents or articles highlighting interventions against COVID-19 implemented by African governments, local health authorities or nongovernmental health organizations. We also relied on our own personal experiences and perspectives. Considering that the response to the epidemic has been very variable from country to country, we agreed to focus our analysis from reports emanating from Uganda, Kenya, Rwanda, Cameroon, Zambia, South Africa and Botswana, countries that were represented among us. An extensive review of the steps taken in preparedness and response to COVID-19 guided by a representative from each of the seven SSA countries was conducted and a conclusive report encompassing our perspectives was generated.

### Sub-Saharan Africa preparedness for COVID-19

Since the beginning of the COVID-19 pandemic, the world’s top priority was to contain the spread of SARS-CoV-2, to reduce disease fatalities and to limit the patient burden on health systems. Despite the uncertainty and unanswered questions around the management of the newly emergent SARS-CoV-2 infection, African countries joined the global effort to battle the COVID-19 pandemic as we outline in detail below. It has been reported that the experiences of SSA countries in handling ongoing outbreaks and managing infectious diseases such as Ebola, tuberculosis, malaria and HIV came in handy in the fight of COVID-19 ^17– 19^. Pre-existing emergency plans on public health interventions, community engagement programs and the work force composed of emergency medical experts and trained health care workers were quickly re-directed to ensure a fast response to COVID-19 ^17– 21^.

As early as January 2020, prior to the identification of the first case of SARS-CoV-2 infection on the African continent on February 14 ^th^ 2020 ^3^, African countries had already initiated public engagement conversations to inform the population about the new pandemic ^22– 28^. Public notices about COVID-19 were issued between January and March in the seven SSA countries that we assessed ^29– 35^. In preparation for possible incoming cases the Ministries of Health worked with local health authorities to designate medical teams, testing laboratories and clinical facilities for isolation and care of COVID-19 patients ^25, 36– 41^. Through media communication, health authorities addressed the new threat, communicating the signs and symptoms of SARS-CoV-2 infection, its mode of transmission as well as the preventive measures and safety guidelines such as hand and respiratory hygiene and social distancing according to WHO guidelines and recommendations ^24, 42– 48^. Some of the countries that were considered in this analysis such as Rwanda and Kenya quickly instituted widespread hand washing stations or used hand sanitizers in public places such as bus stations and restaurant entrances ^49– 51^. Health communication about COVID-19 was made easier by the use of social media platforms such as WhatsApp, Facebook or Twitter, whereas web-based chat or a hotline number that could be contacted for information and inquiries about COVID-19 were made available to the public in all the seven countries ^23, 28, 52– 57^. Furthermore, in an effort guided by Africa CDC to strengthen the emergency response to COVID-19, the readiness of African countries to handle the new disease was assessed and training of health care workers and lab technologists on diagnosis and management of SARS-CoV-2 infection was conducted ^22^.

The majority of African countries lack specialized medical capacity that is critical for handling severe cases of COVID-19, such as intensive care unit (ICU) beds ^11, 58^ and mechanical ventilators ^59^. Therefore, the main priority on the African continent was to contain the infection, initiate immediate testing for suspected cases and to start medical intervention prior to development or progression to severe clinical disease. The countries we assessed focused their efforts on prevention, early identification of new infections and mitigating mass spread of the virus by quickly tracing case contacts based on the available information. All seven countries initiated border screening at ports of entry by March 2020 and a 14-day self-quarantine was recommended for all incoming travellers ^53, 60– 65^.

Although as a result of few or unavailable laboratory technology, some SSA countries could not test for COVID-19 locally, the early established collaborative model among African countries, coordinated by the Africa CDC, increased testing capacity across the continent ^22, 66, 67^ as an example early samples of suspected COVID-19 cases from the Central African Republic were shipped to Rwanda for testing until the local capacity became available. Nonetheless, testing constraints still remain in most countries ^68^, and therefore testing priority was given to most at risk persons such as returning travellers, or the people who have been in contact with confirmed cases, identified through contact tracing by health care workers. All the seven countries have initiated early testing of suspected cases and established designated facilities for testing and clinical care of COVID-19 patients ^30, 36– 41^.

### Sub-Saharan Africa response to COVID-19

The early implementation of COVID-19 preventive measures delayed the rapid spread of the virus within the African population, but these procedures could not completely halt the spread of the virus in all seven countries. Soon after each country had identified the first case of COVID-19, new infections were reported, with the majority related to returnee travellers or contacts of index COVID-19 cases. To reduce the risk of imported cases, these countries, with the exception of Zambia, swiftly closed borders, shut airports and reduced incoming travellers to essential workers and returning residents ^27, 62– 65, 69– 72^. In order to mitigate further spread of the disease, individuals diagnosed with COVID-19 were admitted in designated isolation areas for care and medical assistance while case contact tracing was immediately initiated. Additionally, mass gatherings and non-essential travels were prohibited, government and private business staff were encouraged to work from home and schools were closed ^65, 71– 77^. With the exception of Zambia, the assessed SSA countries implemented a dusk to dawn curfew and nation-wide lockdown to enforce social distancing measures, limiting movements to essential service providers ^71, 78– 83^. These measures were mainly put in place to prevent large volumes of new infections that would result in a high demand for hospital services, potentially leading to overwhelming of the fragile medical infrastructure ^84, 85^.

Contrary to what was initially expected, the spread of SARS-CoV-2 has been relatively slower in Africa ^12^, and COVID-19 infections have been generally mild to moderate, leading to more recoveries and lower fatality rates in the seven SSA countries ^86^ compared to Western countries ^6^. It should also be mentioned that this pandemic started earlier on the other continents, suggesting that it may be too early for SSA to celebrate its relative success as Africa may have not yet faced the highest phase of the disease. However, a comparison of the early phases of the pandemic in some African and European countries has shown a positive impact of early interventions initiated by SSA countries resulting in distinct disease trajectories, For example a comparison of the infection dynamics in the United States, United Kingdom, Italy and Spain vs South Africa and Cameroon has shown a continual exponential peak in non-African countries but slow and gradual increase in both of the SSA countries ^87^.

Our analysis suggests that early initiation of preventive measures, a faster response by timely testing of suspected cases and immediate contact tracing done by SSA countries has mitigated a faster and more extensive spread of the virus in the population. Additionally, a contemporary warm climate may have impacted the dynamics of the SARS-CoV-2 transmission in these countries ^88– 90^. We posit that the predominantly young demography could be a contributing factor to a mild disease and low case fatality observed in Africa ^20, 91^. Furthermore, there are suggestions that cross reactive-immunity resulting from previous infections that are predominant in the region or the universal BCG vaccine policy ^92^, widely recommended for infants vaccination in the assessed countries, may have offered some health benefits such as enhanced lung cells immunity against infections contributing to better clinical outcome of the disease. However, these observations have not yet been confirmed by rigorous evaluations. Altogether, the prevention programs that were put in place and the early response implemented by SSA countries may have mitigated the widespread dissemination of the SARS-CoV-2 virus and fatality due to COVID-19 in SSA countries ^69, 93^. The interventions implemented in all seven SSA for prevention and control of the COVID-19 are summarized in Table 1.

**Table 1.** Summary of interventions implemented for prevention and response to COVID-19.

Preparedness and preventive measures (January-March 2020)							
**Strengthening** **medical capacity** **and testing** **technology.**	In collaboration with WHO, Africa CDC and member states, the following steps were taken: • January 27 ^th^: Africa CDC activated its emergency operations center incident Management system (IMS) for the 2019 n-CoV outbreak. • Procurement of SARS-CoV-2 testing kits • Establishment of collaborative model among African countries, setting up a specimen referral system. • Deployment of health experts, training and technical support of staff from risky areas such as airports. • Development of informational materials on the infection. • Development/strengthening capacity of local health facilities. • Timely communication and weekly updates on high priority areas for coronavirus control. • Avail scientific documentation and references on the new coronavirus.						
	• Ministries of Health and authorities from all seven countries designated a medical team and indicated health facilities for testing and clinical care of COVID-19 patients.						
**Public engagement** **and Educational** **Sessions**	• Health authorities issued public information regarding the new disease, signs and symptoms, health precautions and communicated the WHO/ country specific safety guidelines. • Each country issued an official communication independently on different days as shown below:						
	**Kenya**	**South Africa**	**Cameroon**	**Rwanda**	**Botswana**	**Uganda**	**Zambia**
	February 2 ^nd^	March 7 ^th^	January 28 ^th^	January 21 ^st^	March 16 ^th^	March 2 ^nd^	March 25 ^th^
	• Rwanda and Kenya instituted widespread hand washing stations and used hand sanitizers in public places such as bus stations and restaurant entrances. • Mass media use for awareness and health promotion. • Social media technology facilitated communication about COVID-19 between health care workers and the public. • Each country issued a hotline number specific for COVID-19 information/response.						
**Training health care** **workers and lab** **technologists**	• February 6–7 ^th^ effort guided by Africa CDC for Strengthening the emergence response to COVID-19 by training African laboratories and clinicians on diagnosis and management of SARS-CoV-2 infection was conducted. • Upon training completion, documentation about the critical steps of the management of SARS-CoV-2 infection developed by health authorities in each country was made available to health care workers and posted on the official website.						
January 30 ^th^, 2020: WHO declares COVID-19 a Public health emergency of International concern							
February 14 ^th^, 2020: First case of COVID-19 reported on the African continent (Egypt)							
March 11 ^th^, 2020: WHO declares COVID-19 a pandemic.							
Responsive measures (April-May 2020)							
All the seven sub-Saharan African countries implemented similar responses to COVID-19 except Zambia that didn’t close borders or enforce a national lockdown. The different interventions and their implementation date in each country are presented below: **1**. Border closure for non-citizen and non-essential workers. (Except for Zambia) **2**. COVID-19 screening at port of entry for all seven countries **3**. 14-dayself-quarantine recommended for all incoming travellers. **4**. Isolation of COVID-19 patients at designated facilities and close medical monitoring **5**. Immediate contact tracing and testing. **6**. Prohibit mass gathering and non-essential travels inside the country **7**. Recommendation to work from home for private and government institutions **8**. Schools closure **9**. Dusk to dawn curfew and a national lockdown (Except Zambia)							
**Country**	**Kenya**	**South Africa**	**Cameroon**	**Rwanda**	**Botswana**	**Uganda**	**Zambia**
First positive case	March 12 ^th^	March 5 ^th^	March 6 ^th^	March 14 ^th^	March 30 ^th^	March 21 ^st^	March 18 ^th^
**1–3**	March 15 ^th^	March 16 ^th^	March 18 ^th^	March 3 ^rd^	March 24 ^th^	March 22 ^nd^	February 21 ^st^
**6–8**	March 15 ^th^	March 15 ^th^	March 18 ^th^	March 21 ^st^	March 16 ^th^	March 25 ^th^	March 26 ^th^
**9**	March 28 ^th^ April 6 ^th^	March 27 ^th^	March 18 ^th^	March 28 ^th^	April 2 ^nd^	March 30 ^th^	Not done
**Note1**: Only screening at the port of entry and self-quarantine were implemented in Zambia. **Note2**: A curfew was first implemented in Kenya in March 28 ^th^ followed by a national lockdown in April 6 ^th^ 2020							

### Biomedical, sociocultural and economic challenges met in the course of COVID-19 pandemic

The ability to coordinate a rapid response to COVID-19 by African governments, guided by health and scientific experts in the assessed countries, has shown the continent’s strengths and the expertise to tackle health threats like the COVID-19. However, the main challenges in SSA countries such as poor infrastructure ^94^, clusters of high density populations ^95^, highest global burden of infectious diseases ^96^, and low GDP per capita ^97, 98^ have impacted the sustainability of these interventions resulting in early ease of the key measures to prevent other consequences unrelated to the pandemic. Details about the key challenges are summarized in Table 2.

**Table 2.** Biomedical, Sociocultural and economic challenges of outbreak control.

**CATEGORY**	**Type of challenge encountered**	**Consequences on both preparedness and response to COVID-19 pandemic**
**Biomedical**	Limited capacity for epidemiological techniques such as mathematical modeling to guide the decision making in response to the outbreak, particularly in localised settings.	• Insufficient scientific references to guide the response to the outbreak.
	Insufficient medical infrastructure including laboratory technology, (Example: RT-PCR testing labs)/medical capacity such as ICU services, mechanical ventilators.	• Inability/reduced capacity to perform the required tests locally • Delays in availability of test results • Reduced patient safety that may result in life losses due to unavailability of required medical procedures.
	Shortage of medical supplies and PPE (priority given to COVID-19 medical and research activities)	• Increased risk of infections among health care workers • Interruption/delay of non-COVID-19 related research activities.
	Insufficient testing capacity	• Inability to attain the testing level needed for adequate disease surveillance and control. • Delayed testing that may result in increased disease spread due to late detection of COVID-19 cases.
	Shortage of medical/research and clinical laboratory personnel and space	• Overworked medical personnel • Focused medical attention to COVID-19 delaying non-essential medical services during the pandemic such as the recommended regular medical check-ups and non-life-threatening interventions. • Interruption or delay of non-COVID-19 related medical/research activities (Example: minor/elective surgeries).
	Lack of local biotech capacity to conduct advanced biomedical research studies such as transmissibility of SARS-CoV-2 in the African climate conditions, antibody-based therapy, or vaccine and treatment research in the African population.	• Relying on responses from countries that have the capacity to create solutions. • Unavailability of accurate information relevant to the local context that is important for development of adequate preventive measures.
**Sociocultural**	Interruption of school programs and unavailability of remote education technology	• Delays in completion of school programs
	Structure of the markets, social aspect of the population and the culture	• Difficulties to practice social distancing in the communities
	Science is misunderstood, misinformation about the consequences of the safety recommendations (Some interventions being termed harmful or unethical among some communities. Ex: some have suggested that wearing a mask is detrimental to health due to carbon dioxide poisoning ^103^, misleading myths and numerous faith-related rumors whereby some religious leaders have spread wrong information that they are able to cure COVID-19 ^104^.)	• Mistrust of health care systems • Failure to comply with safety procedures
**Economic**	Insufficient funds	• Limited procurement capacity, • Difficulty to expand existing services or scale up to new available technology.
	Borders closure and reduced frequency of international trade	• Delay of transport of essential materials that are initially imported (Example shortage of infant vaccines, anesthesia used for minor surgeries or dentistry). • Increased cost for medical supplies and imported food items • Unavailability of needed materials locally
	Interrupted supply chain due to market scarcity/ priority given to non- African countries	• Incapacity to obtain suppliers for the African market even when there are available funds. • Shortage of frequently used reagents that need to be imported.
	Poor infrastructure, poverty, informal housing and high population density	• Increased risk to get the infection due to unavailability of essential sanitary services • Nearly impossible to comply with social distancing • Hardly able to implement safety measures
	Reduced job security due to lockdown measures	• Increased unemployment during COVID-19 pandemic • Loss of income for most of the families who depend on casual labor, informal market that have been severely affected by the lockdown.
	Lockdown resulting in reduced movements between cities, unavailability of public transportation and discontinued non-essential work activities including stopping work for researchers working on non COVID-19 projects	• Interruption of pre-existing programs (Ex: HIV prevention programs such as PrEP, ART treatment, TB programs, cancer, maternal health care or non-life- threatening surgeries), • Ironically, the emergence of a new virus has prevented virologists to go to the lab!
	Lack of income due to discontinued earning activities, inability to buy food leading to starvation	• Countries unplanned mobilization of emergency fund to feed poor families. • Early ease of the lockdown that may result in new infections • Re-opening work activities to avoid hunger related deaths.

The main challenges that Africa faces in the response to COVID-19 pertain to lack of local biotechnological production and limited research capacity or expertise in speciality fields, thus making African countries unable to conduct sufficient testing and focused research studies related to disease transmissibility, vaccine or cure research relevant to the local context. Many African countries are relying on equipment and reagents imported from outside the continent. Although, most African countries already have the equipment footprint for COVID-19 tests from the key manufacturers Abbott, Cepheid and Roche, testing capacity is hindered by the inability to rapidly expand the technological capacity, limited funds and more recently by the export restrictions imposed on COVID-19-related supplies ^99^. It is heartening to note that countries such as Senegal and South Africa have initiated programmes to locally develop reagents and PPE ^100– 102^. The shift in focus to production of COVID-19-specific items threatens the pre-existing line products needed for other infections such as HIV and TB. For example procurement of COVID-19 supplies has hampered the existing unstable supply chain for routine medical supplies that are essential for the management of other medical conditions (e.g. antiretroviral therapy, tuberculosis and hepatitis drugs, infant vaccines, anesthesia needed for surgeries and dental treatments, test kits, laboratory reagents and consumables used for other medical/research activities).

The mode of transmission of SARS-CoV-2 has led to enforcement of social distancing measures by restrictions of mass gatherings and a national lockdown in six of the seven countries that we assessed ^62– 65, 71, 72, 74– 77^. This method of prevention has specifically disrupted the school programmes and created economic crises that resulted in hunger ^105^ and other hardships for the large SSA population who depend on casual labor and rely on daily income ^7^. The fragile health systems coupled with lockdown measures have inadvertently reduced access to health care for non-emergency and other pre-existing medical conditions. For example with a re-prioritisation of human resources, it is estimated that an additional 6.5 million TB cases will occur over the next five years ^106^. Although the impact on other diseases such as HIV and malaria has not been assessed, it is likely that the COVID-19 pandemic will set back some of the gains made in the countries’ responses to these killer diseases.

### Recommended solutions to bridge the gaps for improved outbreak preparedness and response

**Figure 1.**
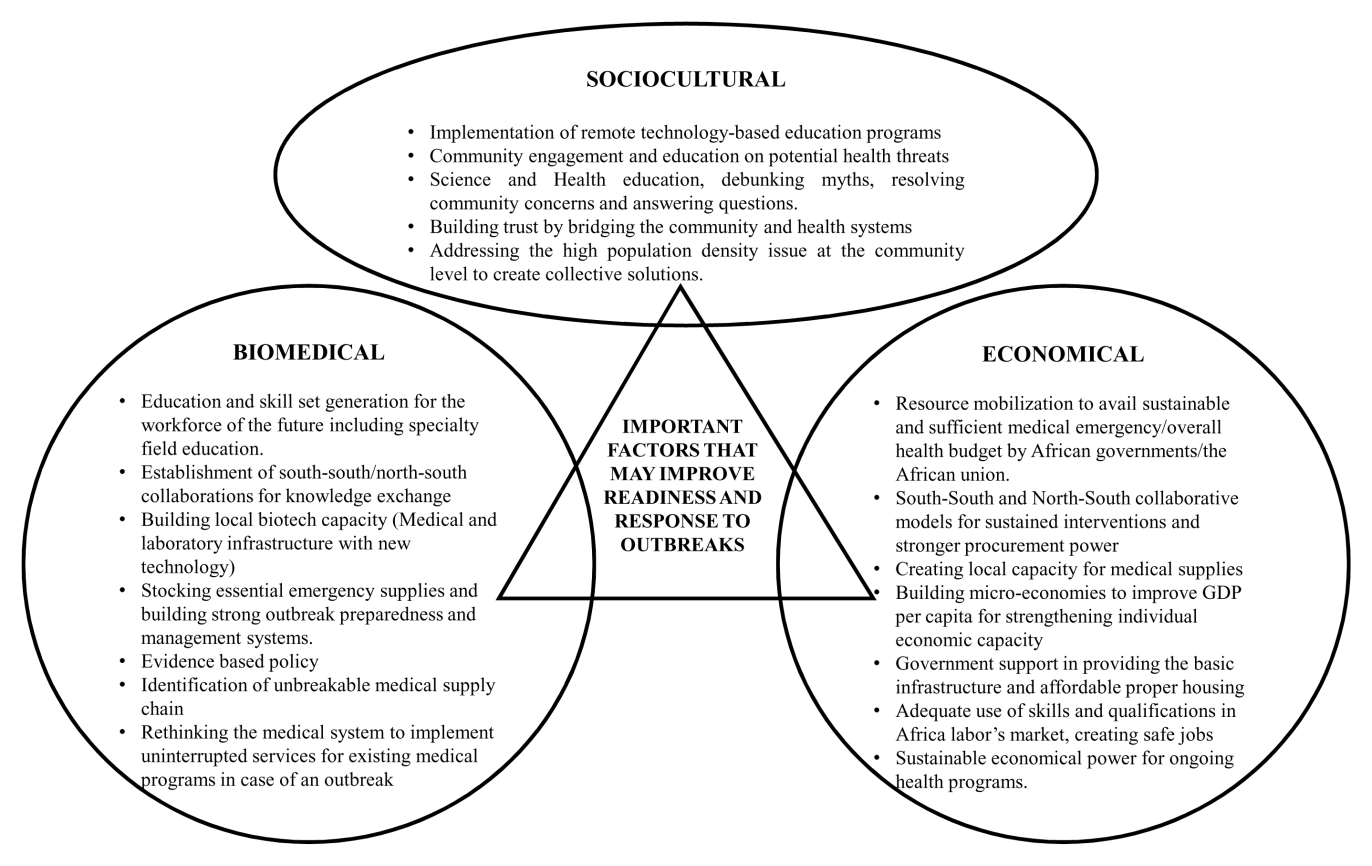
Recommended solutions for improved preparedness and response to future outbreaks.

Based on our assessment of the challenges and the gaps that were found in the approach used by the seven SSA to prevent/respond to COVID-19 outbreak, we summarized the potential solutions in Figure 1. We classified these into three interconnected categories of biomedical, sociocultural and economic aspects that we recommended to help improve the preparedness and response to future outbreaks.

In response to the current COVID-19 pandemic, Africa CDC has started planning for the coordination of a centralised procurement system to reach the target of 55 million tests across the continent. Tens of thousands of test kits, PPE and thermometers have already been distributed to countries through a donation from the Jack Ma Foundation ^99^. However, sustainability of a strong supply chain requires African governments to mobilize resources and to avail funding for health emergency response and research development on the continent including funded education in speciality fields. This will allow the countries to generate adequate interventions and to maintain a rapid response to outbreaks without overreliance on expertise from non-African countries and urgent importation of supplies. SSA countries should leverage novel medical/research capacity upon the existing structures that were put in place over many decades of fighting other public health threats such as Ebola, HIV, TB and malaria. The establishment of a network of multidisciplinary health care workers competent in various tasks such as community education and testing, in a multi-disease focused approach would allow management of staff shortage rather than having to prioritize the new life-threatening disease over those that were already prevailing on the continent.

### Limitations

Some of the limitations encountered during the development of this assessment were mainly related to the lack of sufficient documentation to address the actual reality in SSA countries, such as the status of health systems or informal housing structure. Documentation on physical capacity on the African ground is needed. Some essential documents lacked the important information such as the date signed and released. Improvement on good record keeping especially for health data of this kind is essential for future references.

Our assessment was not designed to demonstrate with certitude that the implemented interventions were directly linked to the number of infections or COVID-19 deaths in the assessed SSA countries. Clinical and biomedical research studies may be more appropriate to confirm these observations. Further, systematic reviews of effects would be informative. Nevertheless, we believe that our unique perspective on the SSA countries preparedness and response to a great health threat such as the COVID-19 pandemic has provided a valuable contribution to the future interventions.

## Conclusion

We assessed the Sub-Saharan Africa preparedness and response to COVID-19. Based on an extensive review of the available scientific publications, the government technical reports and the announcements released by governmental and non-governmental health organizations as well as our personal experiences as workers on the COVID-19 battlefield in SSA countries. This assessment was conducted during the first three month of the COVID-19 pandemic before the virus spread widely in SSA and our conclusions were drawn based on statistical information on the disease presented in this study. We documented the preventive measures and the response put in place to contain the SARS-CoV-2 in seven SSA countries including Uganda, Kenya, Rwanda, Cameroon, Zambia, South Africa and Botswana. We have shown the strengths of early initiated interventions that may have contributed to modest and slower dynamics of COVID-19 in SSA countries. To prepare for the fight against the COVID-19 pandemic, the countries of the sub-Saharan Africa assessed have strengthened their medical capacities by rapidly introducing screening techniques for SARS-CoV-2 and training health care workers in the management of the new disease. Public engagement efforts and information sessions were launched. In response to the COVID-19 pandemic these countries closed their borders and airports, implemented screening for SARS-CoV-2 at port of entry, and introduced a mandatory 14-day quarantine for returning travellers. These SSA countries issued national guidelines on recommended safety measures, initiated immediate contact tracing, prevented mass gatherings, instituted national lockdown and curfew, closed schools and urged the private sector and the government personnel to work from home. It was noted that Zambia, which did not fully implement the interventions described in this study as well as other countries not included in our review that have struggled to put into action public health interventions ^107, 108^, reported no significant increases in deaths compared to countries with more robust responses reported here. There, consistent with the recent study by M. Njenga *et al*. ^109^ which investigated the causes of low morbidity and low mortality of COVID-19 in African countries, we also suggest that other factors such as warm climate, young population, pre-existing cross reactive immunity may have considerably contributed to the evolution of the COVID-19 pandemic in SSA countries. It should be noted that implementation of the public health interventions for preparedness and response to the COVID-19 pandemic in sub-Saharan Africa faced many challenges. We discussed the need for scientific research and evidence-based responses that considered Africa’s unique healthcare, sociocultural and economic challenges. Overall, the assessed countries lacked the local biotechnological capacity for the production of biomedical supplies ^99^, they had a limited workforce with the expertise to specifically address the pandemic using evidence ^110^, there was lack of community trust ^103^, poor infrastructure ^11^, inability to manage remote education programs ^111^, failure to maintain the measures implemented due to the population economic instability ^7, 8^ and insufficient funds ^10^. Formal studies of the extent of these challenges and how to address them in the future will be required. While efforts to bridge some of the gaps have been initiated, we recommend that SSA countries develop continued funding streams to support these initiatives as well to increase south to south/north- south collaborations to enhance the capacity of the existing health systems. Therefore, if these problems are addressed in a timely manner, there is no doubt that in the next five years SSA countries will have developed a reliable-strong health system to prevent the newly emerging viral infections to spread at a large scale.

## Data availability

No data are associated with this article.
