# Supplementary material for: Sub-Saharan Africa preparedness and response to the COVID-19 pandemic: A perspective of early career African scientists
Source: Wellcome Open Res. 2020 Dec 10;5:163. Originally published 2020 Jul 8. [Version 3] doi: 10.12688/wellcomeopenres.16070.3 (PMC7499400; doi:10.12688/wellcomeopenres.16070.3)
Supplement: Peer review history

# Peer review: Reviewer response for version 2

**DOI:** 10.21956/wellcomeopenres.17918.r40487

**Published:** 2020-09-22

**Authors:** Kondwani C. Jambo

**Affiliations:** 1 Liverpool School of Tropical Medicine (LSTM), Liverpool, UK

The authors have adequately addressed my suggestions. I have no further comments.

Does the article adequately reference differing views and opinions?

Partly

Are all factual statements correct, and are statements and arguments made adequately supported by citations?

Yes

Is the Open Letter written in accessible language?

Yes

Where applicable, are recommendations and next steps explained clearly for others to follow?

Yes

Is the rationale for the Open Letter provided in sufficient detail?

Yes

Reviewer Expertise:

Immunology

I confirm that I have read this submission and believe that I have an appropriate level of expertise to confirm that it is of an acceptable scientific standard.

# Peer review: Reviewer response for version 2

**DOI:** 10.21956/wellcomeopenres.17918.r40486

**Published:** 2020-09-17

**Authors:** Charles Shey Wiysonge

**Affiliations:** 1 Cochrane South Africa, South African Medical Research Council, Cape Town, South Africa

Gisele Umviligihozo and colleagues have responded adequately to my comments and suggestions. I have only one discretionary comment at this stage. The authors refer to a "global pandemic" in a few places. Since a pandemic is always global, they should delete the word "global".

Does the article adequately reference differing views and opinions?

Yes

Are all factual statements correct, and are statements and arguments made adequately supported by citations?

Partly

Is the Open Letter written in accessible language?

Yes

Where applicable, are recommendations and next steps explained clearly for others to follow?

Yes

Is the rationale for the Open Letter provided in sufficient detail?

Yes

Reviewer Expertise:

Systematic reviews and meta-analysis, implementation science, evidence-informed policymaking, vaccinology.

I confirm that I have read this submission and believe that I have an appropriate level of expertise to confirm that it is of an acceptable scientific standard.

# Response

**Published:** 2020-09-23

**Authors:** Gisele Umviligihozo

**Affiliations:** Simon Fraser Univeristy, Canada

We thank the reviewer for his positive response, we agree with his comment to replace “global pandemic” with “pandemic”;  this correction has been made in the introduction and in Table 2.

# Peer review: Reviewer response for version 1

**DOI:** 10.21956/wellcomeopenres.17632.r39736

**Published:** 2020-08-18

**Authors:** Kondwani C. Jambo

**Affiliations:** 1 Liverpool School of Tropical Medicine (LSTM), Liverpool, UK

This is an open letter by Umviligihozo *et al. *that aims at documenting the public health interventions against COVID-19 implemented in seven sub-Saharan African countries. Overall, the paper is well written and covers the most pertinent issues on the topic. It highlights the diverse implementation strategies employed by the seven SSA countries but notes the relative similar low mortality experienced in SSA. 

 However, the article could do with a bit of balancing of viewpoints, especially discussing why despite the relatively different implementation strategies of pubic health measures the mortality is relatively low but similar among the SSA countries. In countries like Malawi (not discussed in the article), that have struggled to implement public health interventions, the mortality has not been very different from the other SSA countries. Could this observation mean that it is not necessarily the public health interventions that have driven the low mortality in SSA but potentially other factors? The authors have speculated that other factors including population demographics, cross-reactive immunity, and climate could have contributed to the less severe outcomes, but this has been relegated to a short paragraph. In its current state, the article puts a lot of weight on public health interventions but could be strengthened with a bit more discussion on the other factors. 

 The article would also benefit from a discussion on whether the planned public health interventions in the different countries were successfully implemented. Experiences from other countries show that what was planned on paper is not what happened or is happening on the ground. A discussion on this within the article will aid the reader in the interpretation of the findings and recommendations.

 All in all, I commend the authors for a well-thought-out open letter and for their confidence as young scientists in driving opinion in Africa.

Does the article adequately reference differing views and opinions?

Partly

Are all factual statements correct, and are statements and arguments made adequately supported by citations?

Yes

Is the Open Letter written in accessible language?

Yes

Where applicable, are recommendations and next steps explained clearly for others to follow?

Yes

Is the rationale for the Open Letter provided in sufficient detail?

Yes

Reviewer Expertise:

Immunology

I confirm that I have read this submission and believe that I have an appropriate level of expertise to confirm that it is of an acceptable scientific standard.

# Response

**Published:** 2020-09-07

**Authors:** Gisele Umviligihozo

**Affiliations:** Simon Fraser Univeristy, Canada

We thank the reviewer for the constructive comments and important suggestions for improving this article. Our responses are presented below:

 

- We agree with the reviewer that there are aspects of the COVID-19 presentation in sub-Saharan African that remain unresolved and require additional research. However, our goal here was to review that response in selected African countries and this preliminary analysis suggests a robust and effective response, notwithstanding other factors that may have influenced transmission dynamics and mortality. 

 

In response to the reviewer, we have under the conclusion the following clause that highlighted the importance of other factors driving the observations on infection dynamics of the COVID-19. “It was noted that Zambia, which did not fully implement the interventions described in this study as well as other countries not included in our review that have struggled to put into action public health interventions, ^1,2^ reported no significant increases in deaths compared to countries with more robust responses reported here. There, consistent with the recent study by M. Njenga et *al* ^3^ which investigated the causes of low morbidity and low mortality of COVID-19 in African countries, we also suggest that other factors such as a warm climate, young population, pre-existing cross-reactive immunity may have considerably contributed to the evolution of the COVID-19 pandemic in SSA countries.”

 

- We agree with the reviewer that further studies of the efficiency of implementation of public health interventions is required. This was beyond the scope of the current work. 

 

The following summary discussing the challenges faced in the implementation of the interventions described was added to the text. “It should be noted that implementation of the public health interventions for preparedness and response to the COVID-19 pandemic in sub-Saharan Africa faced many challenges. Overall, the assessed countries lacked the local biotechnological capacity for the production of biomedical supplies, ^4^ they had a limited workforce with the expertise to specifically address the pandemic using evidence, ^5^ there was lack of community trust ^6^, poor infrastructure ^7^, inability to manage remote education programs, ^8^ failure to maintain the measures implemented due to the population economic instability ^9,10^ and insufficient funds. ^11^ Formal studies of the extent of these challenges and how to address them in future will be required.”

**References**

1.        Chibwana MG, Jere KC, Kamn’gona R, et al. High SARS-CoV-2 seroprevalence in health care workers but relatively low numbers of 2 deaths in urban Malawi. *COVID-19 SARS-CoV-2 Prepr from medRxiv bioRxiv*. 2020. doi:https://doi.org/10.1101/2020.07.30.20164970

2.        Mudge L. A Perfect Storm Is Brewing in Burundi WHO Experts Expelled, Election Observers Blocked. Human Rights Watch. https://www.hrw.org/news/2020/05/14/perfect-storm-brewing-burundi. Published 2020. Accessed August 25, 2020.

3.        Njenga MK, Dawa J, Nanyingi M, et al. Why is There Low Morbidity and Mortality of COVID-19 in Africa? *Am J Trop Med Hyg*. 2020;103(2):564-569. doi:10.4269/ajtmh.20-0474

4.        Kavanagh MM, Erondu NA, Tomori O, et al. Access to lifesaving medical resources for African countries : COVID-19 testing and response , ethics , and politics. *Lancet*. 2020;6736(20):19-22. doi:10.1016/S0140-6736(20)31093-X

5.        World Health Organization (WHO). Bulletin of the World Health Organization: Promoting evidence-based health care in Africa. https://www.who.int/bulletin/volumes/95/9/17-030917/en/ . Published 2017. Accessed September 5, 2020.

6.        Goodman; J, Carmichael F. Coronavirus: “Deadly masks” claims debunked. BBC News. https://www.bbc.com/news/53108405. Published July 2020. Accessed August 9, 2020.

7.        Murthy S, Leligdowicz A, Adhikari NKJ. Intensive care unit capacity in low-income countries: A systematic review. *PLoS One*. 2015. doi:10.1371/journal.pone.0116949

8.        Human Rights Watch. Impact of Covid-19 on Children’s Education in Africa. https://www.hrw.org/news/2020/08/26/impact-covid-19-childrens-education-africa#_edn1 . Published 2020. Accessed September 5, 2020.

9.        Potts D. The state and the informal in sub-Saharan African urban economies: Revisiting debates on dualism. *Cris States Res Cent Work Pap*. 2007.

10.      Potts D. The urban informal sector in sub-Saharan Africa: From bad to good (and back again?). In: *Development Southern Africa*. ; 2008. doi:10.1080/03768350802090527

11.      Oleribe OO, Momoh J, Uzochukwu BSC, et al. Identifying key challenges facing healthcare systems in Africa and potential solutions. *Int J Gen Med*. 2019. doi:10.2147/IJGM.S223882

# Peer review: Reviewer response for version 1

**DOI:** 10.21956/wellcomeopenres.17632.r39461

**Published:** 2020-08-04

**Authors:** Charles Shey Wiysonge

**Affiliations:** 1 Cochrane South Africa, South African Medical Research Council, Cape Town, South Africa

I read, with keen interest, the article on public health interventions to fight COVID-19 in Africa by Gisele Umviligihozo and her colleagues, all of them early career scientists, who describe themselves as the next generation of African scientific leaders. This portrays a commendable sense of duty.

 The article deserves indexing and I have only minor comments and suggestions for improvement.

 The authors write that this article is “Based on an extensive review of the available scientific publications, the government technical reports and the announcements released by governmental and nongovernmental health organizations …”. However, the authors do not provide a description of the search and selection of the literature used in this article. I suggest that they should provide this information in the article or as a supplementary document.

 The World Health Organization declared Covid-19 a public health emergency of international concern on 30 January 2020 and not in March 2020. Please refer to reference 1 ^1^.

 The authors state that “Notably, there is no significant increase in unexplained pneumonias or deaths recorded on the continent which could possibly indicate the effectiveness of interventions introduced by several African governments”. While I generally agree with this statement, especially given the time they submitted the article, I would like to alert the authors to reports by the South African Medical Research Council of a recent upsurge in weekly deaths from natural causes in South Africa. Please refer to reference 2 ^2^.

 In Table 2, what does the sociocultural challenge “suspicion of harmful and unethical interventions” mean? What are the harmful and unethical interventions and why and who offered the harmful and unethical interventions to whom?

 What are “faith-related rumors”?

Does the article adequately reference differing views and opinions?

Yes

Are all factual statements correct, and are statements and arguments made adequately supported by citations?

Partly

Is the Open Letter written in accessible language?

Yes

Where applicable, are recommendations and next steps explained clearly for others to follow?

Yes

Is the rationale for the Open Letter provided in sufficient detail?

Yes

Reviewer Expertise:

Systematic reviews and meta-analysis, implementation science, evidence-informed policymaking, vaccinology.

I confirm that I have read this submission and believe that I have an appropriate level of expertise to confirm that it is of an acceptable scientific standard, however I have significant reservations, as outlined above.

## References

[1] : WHO Director-General's statement on IHR Emergency Committee on Novel Coronavirus (2019-nCoV). *Date accessed - 3rd August 2020*. Reference source

[2] : Report on Weekly Deaths in South Africa. *Date Accessed - 3rd August 2020*. Reference source

# Response

**Published:** 2020-09-07

**Authors:** Gisele Umviligihozo

**Affiliations:** Simon Fraser Univeristy, Canada

We thank the reviewer for the positive comments and suggestions for improving this article, our responses are as follows:

  

- To elaborate on the search criteria and to describe the research methodology used in the selection of the literature for this article, the following statement under “Assessment and findings” was updated to reflect these steps. “We therefore decided to review articles focusing on SARS-CoV-2 infection dynamics in Africa and the responses that African governments were taking.  Specifically, we used the free search engine PubMed to identify articles that discussed the transmission dynamics of the new SARS-CoV-2 virus, or described the sociocultural, economic and the state of health care systems in Africa and their impact on SARS-CoV-2 transmission. Moreover, we searched for popular media reports and performed internet searches at official websites for any documents or articles highlighting interventions against COVID-19 implemented by African governments, local health authorities or nongovernmental health organizations.  We also relied on our own personal experiences and perspectives.” 

- We agree with the reviewer's comment on this date, a correction has been made to this statement which now reads “COVID-19 was declared a public health emergency of international concern on January 30 ^th^, 2020 and a global pandemic on March 11 ^th^, 2020 by the World Health Organization (WHO). ^1^"

- We agree with the reviewer’s comment on the need to draw the reader’s attention to the changing dynamics of the COVID-19 pandemic in SSA, to address this concern we updated the introduction to include that recent reports from South Africa indicate a surge in weekly deaths from natural causes that may be attributable to COVID-19. ^2^  We also clarified in conclusion that “This assessment was conducted during the first three month of the COVID-19 pandemic before the virus spread widely in SSA and our conclusions were drawn based on statistical information on the disease presented in this study”

- The sociocultural challenges mentioned in Table 2 refer to the misinformation about the consequences of the safety recommendations. As an example, misinformation has led to some interventions being termed harmful or unethical among some communities.  Examples include interventions such as wearing a mask which some have suggested is detrimental to health due to carbon dioxide poisoning, ^3^ numerous faith-related rumors, whereby some religious leaders have spread misleading information that they are able to cure COVID-19 ^4^. Table 2 was updated to clarify these points.

 

**References**

 1.        World Health Organization (WHO). World Health Organization: WHO Director-General’s statement on IHR Emergency Committee on Novel Coronavirus (2019-nCoV). https://www.who.int/dg/speeches/detail/who-director-general-s-statement-on-ihr-emergency-committee-on-novel-coronavirus-(2019-ncov). Published 2020. Accessed August 9, 2020.

 2.        South African Medical Research Council. Report on Weekly Deaths in South Africa.

 3.        Goodman; J, Carmichael F. Coronavirus: “Deadly masks” claims debunked. BBC News. https://www.bbc.com/news/53108405. Published July 2020. Accessed August 9, 2020.

 4.        FNR TIGG. Pastor Who claimed he cured Coronavirus with faith dies from illness. Complex. https://www.complex.com/life/2020/05/pastor-claims-cured-coronavirus-with-faith-dies-from-illness. Published 2020. Accessed August 9, 2020.
